# What If Low Back Pain Is the Most Prevalent Parkinsonism in the World?

**DOI:** 10.3389/fneur.2018.00313

**Published:** 2018-05-02

**Authors:** Jesse V. Jacobs, Sharon M. Henry, Fay B. Horak

**Affiliations:** ^1^Department of Rehabilitation and Movement Science, University of Vermont, Burlington, VT, United States; ^2^Department of Rehabilitation Therapy, University of Vermont Medical Center, Burlington, VT, United States; ^3^Department of Neurology, School of Medicine, Oregon Health & Science University, Veterans Affairs Portland Health Care System, Portland, OR, United States

**Keywords:** Parkinson’s disease, low back pain, posture, anticipatory postural adjustment, postural response, balance

## Abstract

Low back pain (LBP) has a point prevalence of nearly 10% and ranks highest in global disease burden for years lived with disability; Parkinson’s disease (PD) ranks in the top 100 most disabling health conditions for years lost and years lived with disability (1). Recent evidence suggests that people with chronic, recurrent LBP exhibit many postural impairments reminiscent of a neurological postural disorder such as PD. We compare and contrast postural impairments associated with LBP and PD in order to inform treatment strategies for both conditions. The literature suggests that both LBP and PD associate with impaired proprioceptive function, sensory orientation during standing balance, anticipatory postural adjustments, automatic postural responses, and striatal-cortical function. Although postural impairments are similar in nature for LBP and PD, the postural impairments with LBP appear more specific to the trunk than for PD. Likewise, although both health conditions associate with altered striatal-cortical function, the nature of the altered neural structure or function differ for PD and LBP. Due to the high prevalence of LBP associated with PD, focused treatment of LBP in people with PD may render benefit to their postural impairments and disabilities. In addition, LBP would likely benefit from being considered more than just a musculoskeletal injury; as such, clinicians should consider including approaches that address impairments of postural motor control.

## Introduction

Low back pain (LBP) represents one of the most prevalent health conditions worldwide, having a point prevalence of nearly 10% and ranking first in global disease burden for years lived with disability (1). Parkinson’s disease (PD) also represents a significant health concern as the second-most prevalent neurodegenerative disease in older adults and ranking in the top 100 most disabling health conditions for years lost and years lived with disability (1). Although LBP is a musculoskeletal condition and PD is a neurodegenerative condition, both health conditions present with impairments of postural control and associated alterations of central neurophysiology. For both conditions, these postural impairments span multiple domains of postural control, including (a) reduced somatosensory perception and altered somatosensory integration for balance control; (b) excessive axial postural tone and stiffness; (c) delayed and non-specific anticipatory postural adjustments (APAs) to stabilize and facilitate voluntary movement; (d) non-specific and less effectual automatic postural responses (APRs) to external perturbations; and (e) slowness of walking and other activities. Further, altered structure and function of cortex and basal ganglia is evident for both health conditions. The purpose of this perspective paper is to compare and contrast the postural impairments and related changes in neurophysiology associated with LBP and PD and to discuss the potential implications of their shared impairments on the treatment strategies for both health conditions.

## Sensory Acuity, Kinesthesia, and Dynamic Central Sensory Integration

Any type of physical activity optimally requires accurate sensation and perception of one’s own position and movement (i.e., kinesthesia), and both PD and LBP associate with impaired kinesthesia. People with PD exhibit impaired tactile sensation and impaired kinesthesia to detect limb position during active motion as well as to detect passive limb and trunk rotation (2–8). People with LBP exhibit impaired two-point discrimination and can be unable to kinesthetically perceive their lumbar trunk based on body image traces (9). In contrast to the global somatosensory impairment exhibited by people with PD, the impaired tactile discrimination of people with LBP appears to be isolated to the area of the LBP (9). Impaired lumbosacral repositioning accuracy has also been reported for people with LBP (10). Further, similar to the impaired detection of trunk motion exhibited by people with PD, people with LBP exhibit increased thresholds for detecting passive trunk flexion and lateral bending (11). Thus, although the extent of impairment may differ between people with PD versus LBP, both health conditions associate with impaired tactile acuity and kinesthesia.

The act of maintaining standing balance requires integrating visual, somatosensory, and vestibular inputs. The central nervous system must also modulate each modality’s influence on standing balance when transitioning to different sensory conditions. People with PD exhibit an impaired ability to limit postural sway during standing balance when somatosensory input is incongruent with visual and/or vestibular input (12, 13). Likewise, people with LBP also exhibit increased postural sway under conditions in which somatosensory input is incongruent with the other modalities (14). An enhanced use of ankle proprioception and the ankle strategy for postural sway, rather than a flexible control strategy to utilize trunk proprioception and hip motion under challenging conditions, has also been reported for people with LBP (15). These results suggest that, for people with LBP, the postural impairment may be localized to the processing of trunk proprioception and trunk control, with perhaps compensation through enhanced afferent processing and use of the distal limbs for the control of standing postural sway. Thus, people with LBP and PD alike exhibit an impaired ability to modulate the influence of surface somatosensory input in order to maintain standing balance.

## Mechanical Constraint of Rigidity

Rigidity (resistance to passive movement) is one of the cardinal symptoms of PD and can be evident across axial, proximal, and distal body segments. Although largely neural rather than peripheral in its generation (16), the rigidity associated with PD elicits a significant mechanical constraint that associates with impaired gait quality (17), turning (18), standing postural sway (19), and diminished quality of life (20). Direct measurement of axial rigidity by slow, passive trunk or hip rotation in stance demonstrates an increased rigidity with PD that correlates with clinical symptom scores (21) and with difficulty walking or rolling over (18). Thus, rigidity is a pervasive impairment in PD that influences mobility, balance, and daily life.

Axial or spinal-segmental rigidity is also common in people with LBP, and a change in LBP corresponds with a change in axial rigidity, but these results are not always consistent across studies (22). Although not the intent of a study by Cacciatore and colleagues (and therefore not powered to detect group differences), direct measurement of axial rigidity by slow, passive trunk and hip rotation during standing posture has been evaluated in people with and without LBP (23) using the same methods as those of Wright et al. (21) for people with PD. Cacciatore and colleagues reported nearly identical hip torques between a group of 8 people with LBP (mean ± SD = 3.06 ± 2.19) and a group of 15 control subjects without LBP (3.07 ± 1.66), but a statistically non-significant trend for increased trunk torque (6.26 ± 3.61 for LBP versus 5.00 ± 1.80 without LBP). Although requiring further study with a larger sample, the trend for increased mean rigidity with greater inter-individual variability in the group with LBP suggests that some, but not all, individuals with LBP exhibit axial rigidity (22). If the rigidity is evident, however, it is likely specific to the trunk. Therefore, although both LBP and PD have been associated with rigidity, this mechanical constraint is more consistent and pervasive for PD than for LBP.

## Anticipatory Postural Adjustments

Anticipatory postural adjustments represent learned, centrally programmed muscle activations of supporting body segments to counteract anticipated perturbing forces associated with voluntary movement in order to maintain posture and balance (24). Efficient movement thus depends upon appropriate movement-specific timing and amplitude of APAs.

For PD, impaired APAs appear evident across multiple tasks, such as step initiation and arm raising. During step initiation, for example, people with PD exhibit prolonged and diminished APAs that are poorly scaled to initial mechanical constraints (25, 26). During arm raises, people with PD exhibit APAs that can be delayed beyond a time window of anticipatory control prior to movement-related perturbation, and these delayed postural activations are not specific to the movement (27). Thus, PD associates with delayed, diminished, prolonged, and unspecified APAs across tasks that elicit APAs from axial or distal musculature.

For LBP, the primary impairment of the APA appears to be a delay in activation that can extend beyond a window of anticipatory control prior to movement-related perturbation (28, 29). Interestingly, similar to the findings on people with PD, people with LBP also exhibit a delayed APA that is not specific to the requirements of the movement (30). One notable difference, however, is that the impairment is particularly limited to specific axial muscles and can actually be enhanced or earlier in onset at distal muscles (29). Thus, although delayed and contextually non-specific APAs are shared by both PD and LBP, prolonged duration and generalized impairment across body segments appears more evident with PD than with LBP.

## Automatic Postural Responses

The ability to maintain balance and posture in response to an externally induced postural perturbation is also essential for efficiency and safety during daily activity. APRs represent rapid, automatic, but functionally specific responses to postural perturbations in order to maintain posture and balance.

For PD, the APR results in impaired stability marked by greater induced center-of-mass displacement and diminished corrective center-of-pressure displacement (31, 32). PD is also marked by impaired directional specificity of the APR, in which a non-specific stiffening strategy of antagonistic muscle co-contraction is evident (32, 33).

People with LBP exhibit remarkably similar impairments of the APR as people with PD, demonstrating increased center-of-mass displacements (34), muscle co-contraction (35, 36), and impaired directional specificity of the APR (37, 38). As previously described for both quiet stance and the control of the APA, however, people with LBP also exhibit a redistribution of control for the APR via compensation at distal body segments (36, 37, 39) that is not evident with PD. Thus, both health conditions exhibit diminished stability, co-contraction, and directionally non-specific APRs, but the impairment is more pervasive across body segments for PD, whereas the impairment appears localized to the trunk with compensation elsewhere for LBP.

## Bradykinesia

Bradykinesia is a hallmark feature of parkinsonism. For people with PD, bradykinesia can span movements across body segments, such as finger tapping, smiling, and gait (40–42). For PD, bradykinetic gait is marked by slowed gait velocity, decreased step length, step asymmetries, and variability, and recent studies have also identified altered trunk coordination (42–44).

Interestingly, people with LBP also exhibit slowed gait velocity, decreased step length, step asymmetries, and altered trunk coordination (45–47). Further, as with PD, bradykinesia is not isolated to gait for people with LBP, as they also exhibit slowed trunk motion and lifting behaviors (48, 49). Although the extent of bradykinesia with PD appears greater than for people with LBP when evaluating differences compared to matched control subjects, both health conditions share similar characteristics of bradykinesia.

## Associated Neuropathology

Dysfunction of circuits involving the basal ganglia represents a hallmark pathophysiology associated with the development of motor symptoms in PD, which associates with clinical symptom severity, including postural instability and gait disturbance (50). LBP also associates with pathology of the basal ganglia. The transition from acute to chronic LBP associates with diminished striatal gray matter across multiple nuclei as well as with increased functional connectivity between prefrontal cortex and the nucleus accumbens; this increased connectivity also correlated with reported pain intensity (51). In subjects with established chronic LBP, however, there are many associated changes in neural structures and functions that do not necessarily resemble those of PD (52), including non-overlapping regions of diminished cortical gray matter and increased striatal gray matter (53, 54). Thus, corticostriatal pathology may affect both PD and LBP, but the nature of the pathology is quite different.

Beyond the existence of corticostriatal pathology, more specific alterations of cortical neurophysiology during postural tasks are evident with both PD and LBP. As determined by repetitive transcranial magnetic stimulation, the prolonged APA durations of people with PD during step initiation associate with the function of circuits involving the supplementary motor area, and the influence of stimulation at the supplementary motor area on APA duration appears to increase with increasing disease severity (26). Further, prior to initiating an APA for step initiation, greater amplitudes of electroencephalographic (EEG) preparatory cortical potentials associate with increasing disease severity for people with PD (55). With regard to the APR, people with PD exhibit enhanced preparatory EEG potentials, and the modulation of these potentials associates with the extent of APR modulation between conditions of differing perturbation amplitudes (56). During walking, people with PD exhibit enhanced frontal lobe activity (57). In sum, the results suggest an enhanced influence of the cerebral cortex on postural control for people with PD.

People with LBP likewise exhibit evidence of an increased influence of the cerebral cortex on postural control. As determined by transcranial magnetic stimulation, larger areas of the transversus abdominus muscle’s cortical representation correlate with the onset delay of that muscle’s APA activation during an arm-raise task (58). People with LBP also exhibit increased preparatory EEG potentials (29) as well as an increased topographical area of the potentials (59) prior to arm raises that require an APA. Amplitudes of preparatory EEG potentials have also been reported to correlate with APA onset time for subjects with LBP when performing an arm-raise task (59). With regard to the APR, people with LBP exhibit increased amplitudes of evoked EEG potentials in response to postural perturbation, and the amplitude of these enhanced potentials correlated with evoked center-of-mass displacement as well as the subjects’ reported pain-related disability and fear of physical activity (39). Therefore, people with PD and people with LBP exhibit altered cortical functions that significantly correlate with their postural behavior and clinical symptoms, and this altered cortical function suggests an increased influence of the cerebral cortex on postural control for both health conditions.

Given the complex systems that control posture and gait (60), these few neuropathological similarities do not demonstrate that they are necessary and sufficient to produce the shared motor behaviors of LBP and PD. Although isolated characteristics of LBP could also relate to isolated characteristics of other neurological conditions to suggest other mechanisms of neuropathological involvement, we preliminarily argue that the similarities in overall presentation of posture and gait between LBP and PD are greater than for LBP with other neurological conditions (e.g., cerebellar, vestibular, peripheral neuropathy, stroke), which differ in sensory conditions of impaired standing balance, truncal rigidity, the contextual specificity, scaling, and timing of APAs and APRs, as well as parkinsonian versus ataxic, neuropathic, or hemiparetic gait patterns (61–64). Therefore, relating the control of posture and gait of LBP to that of PD appears more robust than other options.

## LBP Confounds PD

Based on the above sections, many shared postural impairments exist for both PD and LBP. These shared impairments are so extensive that PD can be misdiagnosed as LBP (65). It is important to note, however, that LBP confounds PD, because LBP is often coincident with PD. In almost 30% of cases, LBP is an initial presenting symptom of PD (66, 67). In addition, the prevalence of LBP with PD is approximately 60–83% compared to approximately 25% in matched control subjects (68–70). Thus, it is possible that the postural impairments of PD are exacerbated by the coexistence of LBP, and LBP may be exacerbated by the postural impairments of PD.

## Implications for Treatment

Although multidisciplinary treatment strategies are espoused for both PD and LBP (71, 72), the conservative physical treatment of postural impairment differs considerably between these two conditions. First, despite the prevalence of pain with PD, pain is rarely a focus of treatment for people with PD (69, 70). The treatment of motor impairment, however, is more common for people with PD. For example, physical therapy is utilized by about 63% of cases with PD, and the majority of its use is focused on retraining gait, balance, and posture (73). In contrast, physical therapy is utilized by less than 20% of cases with LBP, and its use comprises approximately six visits that prioritize pain management, strength, and flexibility rather than gait, balance, and postural training (74, 75). Treatment outcomes for LBP with this approach have been variable, although the use of motor control retraining hasn’t yet demonstrated superior treatment outcomes to general exercise (76). The lack of superior treatment outcomes for LBP with motor control retraining therapy, however, may be because the treatment does not adhere to principles of motor rehabilitation that have been more thoroughly researched and considered in practice for neurological rehabilitation (77).

Therefore, despite the many shared postural impairments between PD and LBP, as well as the high prevalence of LBP in people with PD, the treatment approaches of these two health conditions are highly divergent. The coexistence of LBP with PD suggests focused management of pain, strength, and flexibility could potentially, at least partially, help alleviate postural impairment with PD. Similarly, the shared motor impairments of LBP to PD suggests that the management of LBP could optimally include a postural motor retraining approach that is of sufficient focus and training exposure that motor patterns can be modified across multiple domains of postural control. Although important to substantiate mechanisms of pathology associated with LBP as an axial parkinsonism of postural tone and dynamic control, that substantiation does not preclude exploring postural motor retraining for LBP as a potential treatment to improve patient outcomes.

## Summary

**Figure 1:**
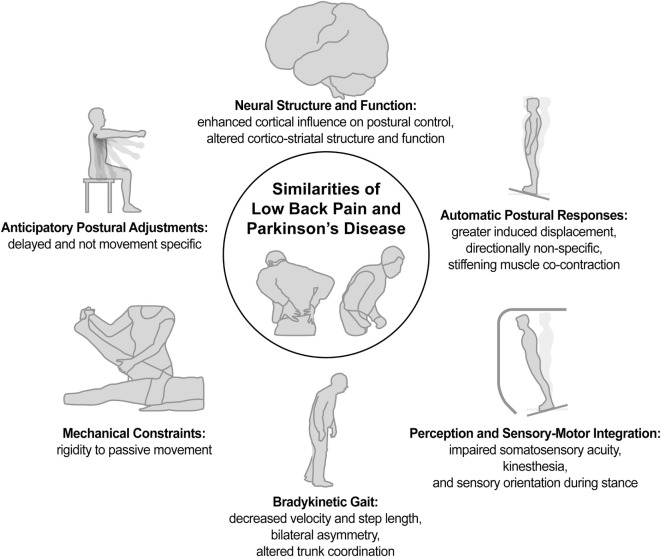
Similarities of low back pain and Parkinson’s disease.

Review of the literature indicates that both PD and LBP exhibit many shared impairments in postural control as well as some similar changes in neural pathology or function (Figure 1). Notably, for LBP (a) the impairments appear less pervasive and more localized to the trunk, (b) the impairments seem less consistent across individuals, and (c) despite some shared characteristics, the neural pathology is holistically of a different nature than for PD. Nevertheless, motor impairments seem more alike than different, suggesting that treatment strategies for LBP could benefit from those provided for PD, and treatment strategies that ameliorate LBP have the potential to benefit the treatment of motor dysfunction and lumbar pain in people with PD. Overall, the similarities of LBP and PD in postural impairment and associated neurophysiology suggest it may not be so implausible to consider LBP as an axial parkinsonism, rendering it the most prevalent parkinsonism in the world.

## Author Contributions

All authors contributed to generating the scientific arguments of this perspective paper. First draft was generated by JJ, and critical review and editing was provided by all other authors.

## Conflict of Interest Statement

FH has an equity interest in APDM, a company that may have commercial interest in the results of this study. This potential conflict of interest has been managed by OHSU and the Portland VA. JJ is employed by Liberty Mutual Insurance. SH declares no commercial or financial relationships that could be construed as a potential conflict of interest.

## References

[1] Stovner LJ Hoff JM Svalheim S Gilhus NE Neurological disorders in the global burden of disease 2010 study. Acta Neurol Scand Suppl (2014) 198:1–6. 10.1111/ane.12229 24588499

[2] Klockgether T Borutta M Rapp H Spieker S Dichgans J. A defect of kinesthesia in Parkinson’s disease. Mov Disord (1995) 10:460–5. 10.1002/mds.870100410 7565827

[3] Demirci M Grill S Mcshane L Hallett M. A mismatch between kinesthetic and visual perception in Parkinson’s disease. Ann Neurol (1997) 41:781–8. 10.1002/ana.410410614 9189039

[4] Sathian K Zangaladze A Green J Vitek JL Delong MR. Tactile spatial acuity and roughness discrimination: impairments due to aging and Parkinson’s disease. Neurology (1997) 49:168–77. 10.1212/WNL.49.1.168 9222186

[5] Adamovich SV Berkinblit MB Hening W Sage J Poizner H. The interaction of visual and proprioceptive inputs in pointing to actual and remembered targets in Parkinson’s disease. Neuroscience (2001) 104:1027–41. 10.1016/S0306-4522(01)00099-9 11457588

[6] Jacobs JV Horak FB. Abnormal proprioceptive-motor integration contributes to hypometric postural responses of subjects with Parkinson’s disease. Neuroscience (2006) 141:999–1009. 10.1016/j.neuroscience.2006.04.014 16713110

[7] Konczak J Corcos DM Horak F Poizner H Shapiro M Tuite P Proprioception and motor control in Parkinson’s disease. J Mot Behav (2009) 41:543–52. 10.3200/35-09-002 19592360

[8] Wright WG Gurfinkel VS King LA Nutt JG Cordo PJ Horak FB. Axial kinesthesia is impaired in Parkinson’s disease: effects of levodopa. Exp Neurol (2010) 225:202–9. 10.1016/j.expneurol.2010.06.016 20599976 PMC3052408

[9] Moseley GL. I can’t find it! Distorted body image and tactile dysfunction in patients with chronic back pain. Pain (2008) 140:239–43. 10.1016/j.pain.2008.08.001 18786763

[10] Brumagne S Cordo P Lysens R Verschueren S Swinnen S The role of paraspinal muscle spindles in lumbosacral position sense in individuals with and without low back pain. Spine (Phila Pa 1976) (2000) 25:989–94. 10.1097/00007632-200004150-00015 10767813

[11] Lee AS Cholewicki J Reeves NP Zazulak BT Mysliwiec LW. Comparison of trunk proprioception between patients with low back pain and healthy controls. Arch Phys Med Rehabil (2010) 91:1327–31. 10.1016/j.apmr.2010.06.004 20801248 PMC4896302

[12] Chong RK Horak FB Frank J Kaye J. Sensory organization for balance: specific deficits in Alzheimer’s but not in Parkinson’s disease. J Gerontol A Biol Sci Med Sci (1999) 54:M122–8. 10.1093/gerona/54.3.M122 10191839

[13] Frenklach A Louie S Koop MM Bronte-Stewart H. Excessive postural sway and the risk of falls at different stages of Parkinson’s disease. Mov Disord (2009) 24:377–85. 10.1002/mds.22358 18972546

[14] Della Volpe R Popa T Ginanneschi F Spidalieri R Mazzocchio R Rossi A. Changes in coordination of postural control during dynamic stance in chronic low back pain patients. Gait Posture (2006) 24:349–55. 10.1016/j.gaitpost.2005.10.009 16311036

[15] Brumagne S Janssens L Knapen S Claeys K Suuden-Johanson E. Persons with recurrent low back pain exhibit a rigid postural control strategy. Eur Spine J (2008) 17:1177–84. 10.1007/s00586-008-0709-7 18594876 PMC2527415

[16] Zetterberg H Frykberg GE Gaverth J Lindberg PG. Neural and nonneural contributions to wrist rigidity in Parkinson’s disease: an explorative study using the NeuroFlexor. Biomed Res Int (2015) 2015:276182. 10.1155/2015/276182 25685778 PMC4320927

[17] Kwon KY Kim M Lee SM Kang SH Lee HM Koh SB. Is reduced arm and leg swing in Parkinson’s disease associated with rigidity or bradykinesia? J Neurol Sci (2014) 341:32–5. 10.1016/j.jns.2014.03.041 24717971

[18] Franzen E Paquette C Gurfinkel VS Cordo PJ Nutt JG Horak FB. Reduced performance in balance, walking and turning tasks is associated with increased neck tone in Parkinson’s disease. Exp Neurol (2009) 219:430–8. 10.1016/j.expneurol.2009.06.013 19573528 PMC2775914

[19] Bartolic A Pirtosek Z Rozman J Ribaric S. Postural stability of Parkinson’s disease patients is improved by decreasing rigidity. Eur J Neurol (2005) 12:156–9. 10.1111/j.1468-1331.2004.00942.x 15679705

[20] Cano-De-La-Cuerda R Vela-Desojo L Miangolarra-Page JC Macias-Macias Y Munoz-Hellin E. Axial rigidity and quality of life in patients with Parkinson’s disease: a preliminary study. Qual Life Res (2011) 20:817–23. 10.1007/s11136-010-9818-y 21170683

[21] Wright WG Gurfinkel VS Nutt J Horak FB Cordo PJ. Axial hypertonicity in Parkinson’s disease: direct measurements of trunk and hip torque. Exp Neurol (2007) 208:38–46. 10.1016/j.expneurol.2007.07.002 17692315 PMC2144734

[22] Wong AY Kawchuk GN The clinical value of assessing lumbar posteroanterior segmental stiffness: a narrative review of manual and instrumented methods. PM R (2016) 9(8):816–30. 10.1016/j.pmrj.2016.12.001 27993736

[23] Cacciatore TW Gurfinkel VS Horak FB Cordo PJ Ames KE. Increased dynamic regulation of postural tone through Alexander technique training. Hum Mov Sci (2011) 30:74–89. 10.1016/j.humov.2010.10.002 21185100 PMC3074502

[24] Massion J Movement, posture and equilibrium: interaction and coordination. Prog Neurobiol (1992) 38:35–56. 10.1016/0301-0082(92)90034-C 1736324

[25] Rocchi L Chiari L Mancini M Carlson-Kuhta P Gross A Horak FB. Step initiation in Parkinson’s disease: influence of initial stance conditions. Neurosci Lett (2006) 406:128–32. 10.1016/j.neulet.2006.07.027 16901637

[26] Jacobs JV Lou JS Kraakevik JA Horak FB. The supplementary motor area contributes to the timing of the anticipatory postural adjustment during step initiation in participants with and without Parkinson’s disease. Neuroscience (2009) 164:877–85. 10.1016/j.neuroscience.2009.08.002 19665521 PMC2762010

[27] Bazalgette D Zattara M Bathien N Bouisset S Rondot P. Postural adjustments associated with rapid voluntary arm movements in patients with Parkinson’s disease. Adv Neurol (1987) 45:371–4. 3825713

[28] Hodges PW Richardson CA. Altered trunk muscle recruitment in people with low back pain with upper limb movement at different speeds. Arch Phys Med Rehabil (1999) 80:1005–12. 10.1016/S0003-9993(99)90052-7 10489000

[29] Sadeghi M Talebian S Olyaei GR Attarbashi Moghadam B. Preparatory brain activity and anticipatory postural adjustments accompanied by externally cued weighted-rapid arm rise task in non-specific chronic low back pain patients and healthy subjects. Springerplus (2016) 5:674. 10.1186/s40064-016-2342-y 27350911 PMC4899386

[30] Lomond KV Jacobs JV Hitt JR Desarno MJ Bunn JY Henry SM Effects of low back pain stabilization or movement system impairment treatments on voluntary postural adjustments: a randomized controlled trial. Spine J (2015) 15:596–606. 10.1016/j.spinee.2014.10.020 25452017 PMC4375040

[31] Horak FB Dimitrova D Nutt JG. Direction-specific postural instability in subjects with Parkinson’s disease. Exp Neurol (2005) 193:504–21. 10.1016/j.expneurol.2004.12.008 15869953

[32] Jacobs JV Dimitrova DM Nutt JG Horak FB. Can stooped posture explain multidirectional postural instability in patients with Parkinson’s disease? Exp Brain Res (2005) 166:78–88. 10.1007/s00221-005-2346-2 16096779 PMC1351284

[33] Dimitrova D Horak FB Nutt JG. Postural muscle responses to multidirectional translations in patients with Parkinson’s disease. J Neurophysiol (2004) 91:489–501. 10.1152/jn.00094.2003 12944541

[34] Henry SM Hitt JR Jones SL Bunn JY. Decreased limits of stability in response to postural perturbations in subjects with low back pain. Clin Biomech (Bristol, Avon) (2006) 21:881–92. 10.1016/j.clinbiomech.2006.04.016 16806618

[35] Radebold A Cholewicki J Panjabi MM Patel TC. Muscle response pattern to sudden trunk loading in healthy individuals and in patients with chronic low back pain. Spine (Phila Pa 1976) (2000) 25:947–54. 10.1097/00007632-200004150-00009 10767807

[36] Jones SL Henry SM Raasch CC Hitt JR Bunn JY. Individuals with non-specific low back pain use a trunk stiffening strategy to maintain upright posture. J Electromyogr Kinesiol (2012) 22:13–20. 10.1016/j.jelekin.2011.10.006 22100719 PMC3246114

[37] Jacobs JV Henry SM Jones SL Hitt JR Bunn JY. A history of low back pain associates with altered electromyographic activation patterns in response to perturbations of standing balance. J Neurophysiol (2011) 106:2506–14. 10.1152/jn.00296.2011 21795622 PMC3214123

[38] Jacobs JV Lomond KV Hitt JR Desarno MJ Bunn JY Henry SM Effects of low back pain and of stabilization or movement-system-impairment treatments on induced postural responses: a planned secondary analysis of a randomised controlled trial. Man Ther (2016) 21:210–9. 10.1016/j.math.2015.08.006 26324322 PMC4713345

[39] Jacobs JV Roy CL Hitt JR Popov RE Henry SM Neural mechanisms and functional correlates of altered postural responses to perturbed standing balance with chronic low back pain. Neuroscience (2016) 339:511–24. 10.1016/j.neuroscience.2016.10.032 27771534 PMC5118100

[40] Marsili L Agostino R Bologna M Belvisi D Palma A Fabbrini G Bradykinesia of posed smiling and voluntary movement of the lower face in Parkinson’s disease. Parkinsonism Relat Disord (2014) 20:370–5. 10.1016/j.parkreldis.2014.01.013 24508573

[41] Bologna M Leodori G Stirpe P Paparella G Colella D Belvisi D Bradykinesia in early and advanced Parkinson’s disease. J Neurol Sci (2016) 369:286–91. 10.1016/j.jns.2016.08.028 27653910

[42] Peterson DS Horak FB. Neural control of walking in people with parkinsonism. Physiology (Bethesda) (2016) 31:95–107. 10.1152/physiol.00034.2015 26889015 PMC4888974

[43] Tan D Danoudis M Mcginley J Morris ME. Relationships between motor aspects of gait impairments and activity limitations in people with Parkinson’s disease: a systematic review. Parkinsonism Relat Disord (2012) 18:117–24. 10.1016/j.parkreldis.2011.07.014 22093237

[44] Horak FB Mancini M. Objective biomarkers of balance and gait for Parkinson’s disease using body-worn sensors. Mov Disord (2013) 28:1544–51. 10.1002/mds.25684 24132842 PMC3927718

[45] Keefe FJ Hill RW. An objective approach to quantifying pain behavior and gait patterns in low back pain patients. Pain (1985) 21:153–61. 10.1016/0304-3959(85)90285-4 3157094

[46] Lamoth CJ Daffertshofer A Meijer OG Beek PJ. How do persons with chronic low back pain speed up and slow down? Trunk-pelvis coordination and lumbar erector spinae activity during gait. Gait Posture (2006) 23:230–9. 10.1016/j.gaitpost.2005.02.006 16399520

[47] Cimolin V Vismara L Galli M Zaina F Negrini S Capodaglio P. Effects of obesity and chronic low back pain on gait. J Neuroeng Rehabil (2011) 8:55. 10.1186/1743-0003-8-55 21943156 PMC3186748

[48] Marras WS Wongsam PE. Flexibility and velocity of the normal and impaired lumbar spine. Arch Phys Med Rehabil (1986) 67:213–7. 2938557

[49] Boston JR Rudy TE Lieber SJ Stacey BR. Measuring treatment effects on repetitive lifting for patients with chronic low back pain: speed, style, and coordination. J Spinal Disord (1995) 8:342–51. 10.1097/00002517-199510000-00002 8563153

[50] Hsiao IT Weng YH Hsieh CJ Lin WY Wey SP Kung MP Correlation of Parkinson disease severity and 18F-DTBZ positron emission tomography. JAMA Neurol (2014) 71:758–66. 10.1001/jamaneurol.2014.290 24756323

[51] Baliki MN Petre B Torbey S Herrmann KM Huang L Schnitzer TJ Corticostriatal functional connectivity predicts transition to chronic back pain. Nat Neurosci (2012) 15:1117–9. 10.1038/nn.3153 22751038 PMC3411898

[52] Kregel J Meeus M Malfliet A Dolphens M Danneels L Nijs J Structural and functional brain abnormalities in chronic low back pain: a systematic review. Semin Arthritis Rheum (2015) 45:229–37. 10.1016/j.semarthrit.2015.05.002 26092329

[53] Schmidt-Wilcke T Leinisch E Ganssbauer S Draganski B Bogdahn U Altmeppen J Affective components and intensity of pain correlate with structural differences in gray matter in chronic back pain patients. Pain (2006) 125:89–97. 10.1016/j.pain.2006.05.004 16750298

[54] Mao C Wei L Zhang Q Liao X Yang X Zhang M. Differences in brain structure in patients with distinct sites of chronic pain: a voxel-based morphometric analysis. Neural Regen Res (2013) 8:2981–90. 10.3969/j.issn.1673-5374.2013.32.001 25206618 PMC4146206

[55] Popov RE Gamache J Hitt JR Boyd JT Jacobs JV Effects of Parkinson’s disease on neural preparation and step initiation in unpredictable conditions. Society for Neuroscience Annual Meeting. San Diego, CA (2016).

[56] Smith BA Jacobs JV Horak FB. Effects of magnitude and magnitude predictability of postural perturbations on preparatory cortical activity in older adults with and without Parkinson’s disease. Exp Brain Res (2012) 222:455–70. 10.1007/s00221-012-3232-3 22936099 PMC3472959

[57] Maidan I Nieuwhof F Bernad-Elazari H Reelick MF Bloem BR Giladi N The role of the frontal lobe in complex walking among patients with Parkinson’s disease and healthy older adults: an fNIRS study. Neurorehabil Neural Repair (2016) 30:963–71. 10.1177/1545968316650426 27221042

[58] Tsao H Galea MP Hodges PW. Reorganization of the motor cortex is associated with postural control deficits in recurrent low back pain. Brain (2008) 131:2161–71. 10.1093/brain/awn154 18669505

[59] Jacobs JV Henry SM Nagle KJ. Low back pain associates with altered activity of the cerebral cortex prior to arm movements that require postural adjustment. Clin Neurophysiol (2010) 121:431–40. 10.1016/j.clinph.2009.11.076 20071225 PMC2822008

[60] Takakusaki K. Functional neuroanatomy for posture and gait control. J Mov Disord (2017) 10:1–17. 10.14802/jmd.16062 28122432 PMC5288669

[61] Morton SM Bastian AJ. Cerebellar control of balance and locomotion. Neuroscientist (2004) 10:247–59. 10.1177/1073858404263517 15155063

[62] Horak FB. Postural compensation for vestibular loss. Ann N Y Acad Sci (2009) 1164:76–81. 10.1111/j.1749-6632.2008.03708.x 19645883 PMC3224857

[63] Beyaert C Vasa R Frykberg GE. Gait post-stroke: pathophysiology and rehabilitation strategies. Neurophysiol Clin (2015) 45:335–55. 10.1016/j.neucli.2015.09.005 26547547

[64] Alam U Riley DR Jugdey RS Azmi S Rajbhandari S D’aout K Diabetic neuropathy and gait: a review. Diabetes Ther (2017) 8:1253–64. 10.1007/s13300-017-0295-y 28864841 PMC5688977

[65] Burton RR. Parkinson’s disease without tremor masquerading as mechanical back pain; a case report. J Can Chiropr Assoc (2008) 52:185–92. 18769602 PMC2528272

[66] Sandyk R Back pain as an early symptom of Parkinson’s disease. S Afr Med J (1982) 61:3. 6460325

[67] Farnikova K Krobot A Kanovsky P. Musculoskeletal problems as an initial manifestation of Parkinson’s disease: a retrospective study. J Neurol Sci (2012) 319:102–4. 10.1016/j.jns.2012.05.002 22656184

[68] Etchepare F Rozenberg S Mirault T Bonnet AM Lecorre C Agid Y Back problems in Parkinson’s disease: an underestimated problem. Joint Bone Spine (2006) 73:298–302. 10.1016/j.jbspin.2005.05.006 16376599

[69] Broetz D Eichner M Gasser T Weller M Steinbach JP. Radicular and nonradicular back pain in Parkinson’s disease: a controlled study. Mov Disord (2007) 22:853–6. 10.1002/mds.21439 17357131

[70] Beiske AG Loge JH Ronningen A Svensson E. Pain in Parkinson’s disease: prevalence and characteristics. Pain (2009) 141:173–7. 10.1016/j.pain.2008.12.004 19100686

[71] Breen AC Van Tulder MW Koes BW Jensen I Reardon R Bronfort G. Mono-disciplinary or multidisciplinary back pain guidelines? How can we achieve a common message in primary care? Eur Spine J (2006) 15:641–7. 10.1007/s00586-005-0883-9 15931509 PMC3489332

[72] Van Der Marck MA Bloem BR. How to organize multispecialty care for patients with Parkinson’s disease. Parkinsonism Relat Disord (2014) 20(Suppl 1):S167–73. 10.1016/S1353-8020(13)70040-3 24262173

[73] Nijkrake MJ Keus SH Oostendorp RA Overeem S Mulleners W Bloem BR Allied health care in Parkinson’s disease: referral, consultation, and professional expertise. Mov Disord (2009) 24:282–6. 10.1002/mds.22377 19170189

[74] Poitras S Blais R Swaine B Rossignol M. Management of work-related low back pain: a population-based survey of physical therapists. Phys Ther (2005) 85:1168–81. 10.1093/ptj/85.11.1168. 16253046

[75] Carey TS Freburger JK Holmes GM Castel L Darter J Agans R A long way to go: practice patterns and evidence in chronic low back pain care. Spine (Phila Pa 1976) (2009) 34:718–24. 10.1097/BRS.0b013e31819792b0 19282797 PMC2664198

[76] Saragiotto BT Maher CG Yamato TP Costa LO Costa LC Ostelo RW Motor control exercise for nonspecific low back pain: a Cochrane review. Spine (Phila Pa 1976) (2016) 41:1284–95. 10.1097/BRS.0000000000001645 27128390

[77] Winstein C Lewthwaite R Blanton SR Wolf LB Wishart L. Infusing motor learning research into neurorehabilitation practice: a historical perspective with case exemplar from the accelerated skill acquisition program. J Neurol Phys Ther (2014) 38:190–200. 10.1097/NPT.0000000000000046 24828523 PMC5348298

